# Examining variable selection methods for the predictive performance of regression models and the proportion of selected variables and selected random variables

**DOI:** 10.1016/j.heliyon.2021.e07356

**Published:** 2021-06-18

**Authors:** Hiromasa Kaneko

**Affiliations:** Department of Applied Chemistry, School of Science and Technology, Meiji University, 1-1-1 Higashi-Mita, Tama-ku, Kawasaki, Kanagawa 214-8571, Japan

**Keywords:** Variable selection, Feature selection, Regression, Predictive accuracy, Interpretability, QSPR, QSAR

## Abstract

The selection of a descriptor, X, is crucial for improving the interpretation and prediction accuracy of a regression model. In this study, the prediction accuracy of models constructed using the selected X was determined and the results of variable selection, according to the number of selected X and number of selected variables that are unrelated to an objective variable, such as activities and properties (y), were investigated to evaluate the variable or feature selection methods. Variable selection methods include least absolute shrinkage and selection operator, genetic algorithm-based partial least squares, genetic algorithm-based support vector regression, and Boruta. Several regression analysis methods were used to test the prediction accuracy of the model constructed using the selected X. The characteristics of each variable selection method were analyzed using eight datasets. The results showed that even when variables unrelated to y were selected by variable selection and the number of unrelated variables was the same as the number of the original variables, a regression model with good accuracy, which ignores the influence of such noise variables, can be constructed by applying various regression analysis methods. Additionally, the variables related to y must not to be deleted. These findings provide a basis for improving the variable selection methods.

## 1 Introduction

In the fields of chemistry and chemical engineering, data-driven molecular design, material design, process design, and process management have become common. In these processes, data are converted to information using databases of molecules, materials, process simulations, and chemical/industrial plants. Knowledge is subsequently extracted from this information. New chemical structures, experimental conditions, and process conditions are then proposed based on the knowledge extracted.

Among the steps involved in creating a numerical model from databases, our focus is on regression analysis. In molecular design, regression models are constructed based on the chemical structures, physical properties, and activities of molecules. Such models are called quantitative structure-activity relationship (QSAR) [1, 2] and quantitative structure–property relationship (QSPR) [3, 4]. In material design, regression models are constructed based on the experimental conditions and material properties [5]. In process design, regression models are constructed based on the conditions of computer simulations and the simulation results. Such models are called surrogate models [6]. In process control, regression models are constructed based on easily measurable process variables and difficult-to-measure process variables. Such models are referred to as soft sensors [7]. It is desirable for each model to have a high prediction accuracy for analysis when new samples are used.

Approaches for constructing regression models include linear and nonlinear regression analysis methods. Linear regression analysis methods include partial least squares (PLS) regression [8], ridge regression (RR), least absolute shrinkage and selection operator (LASSO), and elastic net (EN) [9]. Nonlinear regression analysis methods include support vector regression (SVR) [10], decision tree (DT) [11], random forests (RF) [12], Gaussian process regression (GPR) [10], gradient boosting decision tree (GBDT) [13], extreme gradient boosting (XGBoost) [14], light gradient boosting machine (LightGBM) [15, 16, 17], CatBoost [18, 19], and deep neural networks (DNNs) [20]. As there is no “best” method for regression analysis, when a database is given, training and test data are required to properly train and evaluate a model.

Descriptors, variables, or feature selection techniques are used to construct regression models with high prediction accuracy. It is believed that high-accuracy prediction models are constructed by deleting noise descriptors (X) that are unrelated to the objective variable such as activities and properties (y) and then selecting only the X that are related to y. Examples of variable selection methods include LASSO and genetic algorithm (GA)-based PLS (GAPLS) [21], which are based on the linear relationship between X and y, as well as GA-based SVR (GASVR) [22] and Boruta [23] methods, which are based on the non-linear relationship between X and y. The hybrid feature selection approach involves two steps for selecting the most informative features. The first is a pre-processing step applied to filter out noise, and the second step is a wrapper technique that selects a set of optimum features. Bio-inspired evolutionary methods such as GA [24], ant colony optimization [25], Bat algorithm [26], artificial bee colony [27], and particle swarm optimization [28] have been applied in feature selection [29]. Although numerous variable selection methods have been developed, the evaluation of a variable selection method is primarily based on the prediction accuracy of the regression model constructed based on y and the selected X. For example, X can be selected with the highest r^2^ value; however, the predictive ability of the regression model is poor because the algorithm predicts with a bias to optimize r^2^. Furthermore, although the performance of a regression analysis method varies depending on the dataset, the choice of the regression method used after variable selection is limited. In addition, variable selection enables the creation of a model based on a smaller number of X, and the relationship between y and the selected X can be interpreted to clarify the relationships between chemical structures and activities/properties, depending on the dataset used. However, this does not necessarily mean that such an interpretation is possible with any dataset.

Therefore, not only the prediction accuracy of regression models with the selected X, but also the results of variable selection according to the number of the selected X and the number of selected X that are unrelated to y are discussed in this study. Several regression analysis methods are used to test the prediction accuracy of the model constructed using the selected X. The variable selection methods focused upon in this study include LASSO, GAPLS, GASVR, and Boruta. LASSO is a linear variable selection method that produces analytical solutions and can be applied to QSAR datasets [30, 31, 32] to reduce prediction errors of QSAR models and to increase the robustness of QSAR models. In LASSO, the regression coefficient can become zero, and the corresponding X can be deleted, indicating variable selection. GAPLS is also a linear variable selection method; however, the selected variables depend on random numbers because variable selection is based on a metaheuristic algorithm. GAPLS was applied to QSAR datasets [33, 34] and could efficiently reduce the number of variables. GASVR is a nonlinear variable selection method, and the selected variables depend on random numbers as well as GAPLS. Although GASVR is time-consuming, it can select X considering nonlinear relationships between X and y and can be applied to QSAR datasets [35, 36]. Boruta is also a nonlinear variable selection method that can be applied to QSAR datasets [37, 38, 39]. Although the percentile is a parameter related to the selected variables and 100% is used in principle, a method called r-Boruta was developed to determine this parameter based on a pseudo-correlation in this study.

The purpose of this study is to discuss variable selection methods in terms of the predictive performance of regression models, proportion of selected variables, and proportion of selected random variables in QSAR/QSPR.

Contribution:

1) The number of selected original X and the number of random variables are large in LASSO and small in Boruta. GAPLS, GASVR, and r-Boruta fall between these two methods in terms of these parameters. However, the prediction error of a regression model does not necessarily increase with a larger number of selected random variables.
2) In r-Boruta, the number of selected random variables is larger than that in Boruta, but the prediction error of the regression model tends to be smaller.
3) Even if X unrelated to y is selected by variable selection, a regression model with good accuracy that ignores the influence of such noise variables can be constructed by using various regression analysis methods. It is important that X related to y is not deleted.
4) Although the results of variable selection and mean absolute error (MAE) are stable in both Boruta and r-Boruta, the variability in the MAE is particularly large in methods such as GAPLS and GASVR.
5) As the number of selected X and random variables is small in Boruta, it can be effective for interpreting the results of variable selection. The number of selected X is large in LASSO, and it is difficult to interpret the results of variable selection.

## 2 Method

In this section, we discuss LASSO, GAPLS, GASVR, Boruta, and r-Boruta. Thereafter, we describe the proposed indicators of the variable selection methods.

### 2.1 LASSO

LASSO is a linear regression analysis method that reduces both the sum of squares of errors and the sum of the absolute values of regression coefficients. The regression coefficient is determined by minimizing the following formula:

(1) $$\sum_{i=1}^{m}{\left(y^{\left(i\right)}-x^{\left(i\right)}b\right)}^{2}+\lambda \sum_{j=1}^{n}\left|b_{j}\right|$$

where *m* is the number of samples, *n* is the number of *x* variables, *y*^(*i*)^ and **x**^(*i*)^ ε R^1×*m*^ are the *y* and *x* values in the *i*th sample, respectively, *b*_*j*_ is the *j*th regression coefficient, and *λ* is the hyperparameter. **b** is represented by the following formula.

$$b={\left(\begin{array}{llll} b_{1} & b_{2} & \cdots & b_{n} \end{array}\right)}^{\text{T}}$$

In LASSO, the regression coefficient, *b*_*j*_, can become zero, and the corresponding *x* is deleted.

In this study, *λ* was set to 2^−15^, 2^−14^, …, 2^−2^, 2^−1^ to consider *λ* over a wide range of values, and the value that maximizes the coefficient of determination, r^2^, after 5-fold cross-validation was used. Sckit-learn [40] was used to calculate LASSO.

### 2.2 GAPLS and GASVR

GAPLS and GASVR are methods that select the optimal combination of variables in the PLS and SVR models, respectively, based on the GA. The GA is an optimization methodology that imitates the evolution of organisms through the repetition of selection, crossing, and mutation. In GAPLS, the number of chromosomes, *x,* is given as a sequence of values from 0 to 1. In GASVR, in addition to the GAPLS chromosome, three parameters are added using a Gaussian kernel. The parameters are the regularization parameter (a hyperparameter), tolerance, and Gaussian kernel parameter. We used the r^2^ value after 5-fold cross-validation to determine the closeness of fit. This makes it possible to select a combination of variables that improves the estimation performance of the PLS model with GAPLS and SVR with GASVR.

We used DEAP [41] to calculate the GA and scikit-learn [40] to calculate the PLS and SVR.

### 2.3 Boruta and r-Boruta

Boruta is a variable selection method based on the importance of the variables in the RF. The importance of each *x* is calculated by performing RF after adding the objective variable and unrelated explanatory variables to the dataset. Important variables are selected from the original set of X by comparing the importance level of X. The Boruta algorithm is as follows.

1. Copy the dataset (matrix) for X.
2. In the copied matrix, the sample values are shuffled for each variable. The variables prepared here are referred to as shuffled X. As the values are shuffled according to variables, the shuffled X and y become unrelated.
3. The datasets of the original X and shuffled X are combined before performing the RF with y and calculating the importance of the variables.
4. Although the shuffled X is unrelated to y, as some values are assigned to the shuffled X to denote the importance of the variable, their *p*-percentile is used as the reference value. In general, *p* = 100; in other words, the maximum importance level of the shuffled X is used as the reference value. Among the original X, those for which the importance exceeds that of the reference value are considered hit variables.
5. When steps 3 and 4 are repeated, a two-sided test based on the binomial distribution, which is the binomial test for the statistical significance of deviations from a theoretically expected distribution of observations into two categories, is performed to examine whether the original X is significant, compared with the shuffled X. Here, the significance level is considered to be *α* = 0.05. During the repetitions, the original X, deemed unimportant compared with the shuffled X, are deleted.

We used Python's boruta_py [23] for calculations.

Boruta cannot determine the specific number of selected variables. The *p*-percentile in the Boruta algorithm controls the number of selected variables, and the smaller the *p*, the larger is the number of selected variables. In general, *p* = 100 in Boruta. That is, the maximum variable importance of the shuffled X is used as the standard, but the smaller the number of samples, the greater is the likelihood of the shuffled X being correlated with y accidently. Hence, when the sample size is small, there is a risk of deleting too much X. Therefore, in this study, after a large number of X was generated based on random numbers that follow the standard normal distribution, the correlation coefficients between random X and y were calculated, and 100-fold of their maximum absolute values were regarded as *p*. If *p* in this method is set with a consideration of chance correlation, the over-deletion of X when the sample size is particularly small can be prevented. This approach is called r-Boruta.

### 2.4 Evaluation of indicators of variable selection methods

We evaluated variable selection and feature selection methods based on the following metrics: prediction performance of the regression model constructed based on the selected X, ratio of the number of selected X to the number of the original X, and ratio of the number of selected random variables to the number of the original X. Random X refers to the variables generated based on the random numbers that follow a standard normal distribution. The number of random X is the same as that of the original X.

As the estimation accuracy of regression models varies depending on the method used to construct the model, discussing the prediction accuracy of a regression model based on a single regression analysis method is meaningless. Hence, we considered the following 13 methods of regression analysis: PLS, RR, LASSO, EN, SVR, DT, RF, GPR, GBDT, XGBoost, LightGBM, CatBoost, and DNN.

Note that because SVR uses linear and Gaussian kernels and GPR uses 11 kernel functions including linear kernel, regression models are constructed using a total of 24 methods. After the training data were used to construct the regression model, the test data were used to make predictions, and the MAE was calculated by using the following formula.

(2) $$MAE=\frac{\sum_{i=1}^{m}\left|y^{\left(i\right)}-y_{\text{P}}^{\left(i\right)}\right|}{m}$$

The 10th percentile MAE of the 24 MAEs, that is, the top 10% MAEs, are regarded as the indicators of the prediction performance of the variables selected by variable selection methods.

The ratio of selected X is determined by dividing the number of selected X by the total number of X.

To determine the ratio of the number of selected X, a uniform random number of variables between 0 and 1, equal to the number of original X, was added to the dataset of the original X variables before executing variable selection. The ratio of the number of selected random variables is determined by dividing the number of selected uniform random variables by the number of original X.

In the next section, the results of variable selection by using variable selection methods are evaluated and discussed based on the three aforementioned metrics. The smaller the MAE in the test data, the greater is the prediction performance of the selected variables. Similarly, the smaller the ratio of the number of selected random variables, the easier it is to interpret the results of the variable selection. Small ratios of the number of selected variables and random variables signify that the number of variables unrelated to y is small; this indicates that the prediction accuracy of the regression model may improve. However, if too many variables are deleted, including the important variables related to y, the prediction accuracy of the regression model may deteriorate.

## 3 Results and discussion

To discuss the variable selection models, namely, LASSO, GAPLS, GASVR, Boruta, and r-Boruta, we used datasets of boiling points (BP) [42], solubility in water (logS) [43], pharmacological activity (pIC_50_) [44], and environmental toxicity (pIGC_50_) [45] as a dataset for compounds. Similarly, we used tablet dataset 1 of Shootout2002 [46] (API1), tablet dataset 2 of Shootout2012 [47] (API2), dataset for wheat [48, 49] (Wheat), and dataset for gasoline [50] (Gasoline) as spectral datasets. In the dataset for compounds, RDKit [51] and Mordred [52] were used for the calculation of molecular descriptors. The QSAR dataset of pIC_50_, obtained from an angiotensin-converting enzyme [44], in which activities were spread over a wide range, was analyzed. The activity of molecules against the target was expressed as the logarithm of 50% inhibitory concentration in μmol/m^3^ (pIC_50_). The QSAR dataset of pIGC_50_ was downloaded from the Environmental Toxicity Prediction Challenge 2009 website [45]. This is an online challenge that invites researchers to predict the toxicity of molecules against *T. Pyriformis*, expressed as the logarithm of 50% growth inhibitory concentration in mg/L (pIGC_50_). The datasets are listed in Table 1. The data were randomly split into 70% and 30% for training and testing, respectively. Those X for which the ratio of samples with the same values in the training data accounted for 80% or more were deleted. One of the pairs of X, for which the absolute value of the correlation coefficient was 1, was subsequently deleted.

**Table 1:** Description of the datasets.

Dataset		# of X variables	# of training samples	# of test samples
BP	RDKit	75	206	88
	Mordred	855	206	88
logS	RDKit	94	903	387
	Mordred	800	903	387
pIC_50_	RDKit	102	80	34
	Mordred	1066	80	34
pIGC_50_	RDKit	90	849	364
	Mordred	828	849	364
API1		650	459	196
API2		372	168	68
Wheat		100	366	157
Gasoline		401	42	18

**Figure 1:**
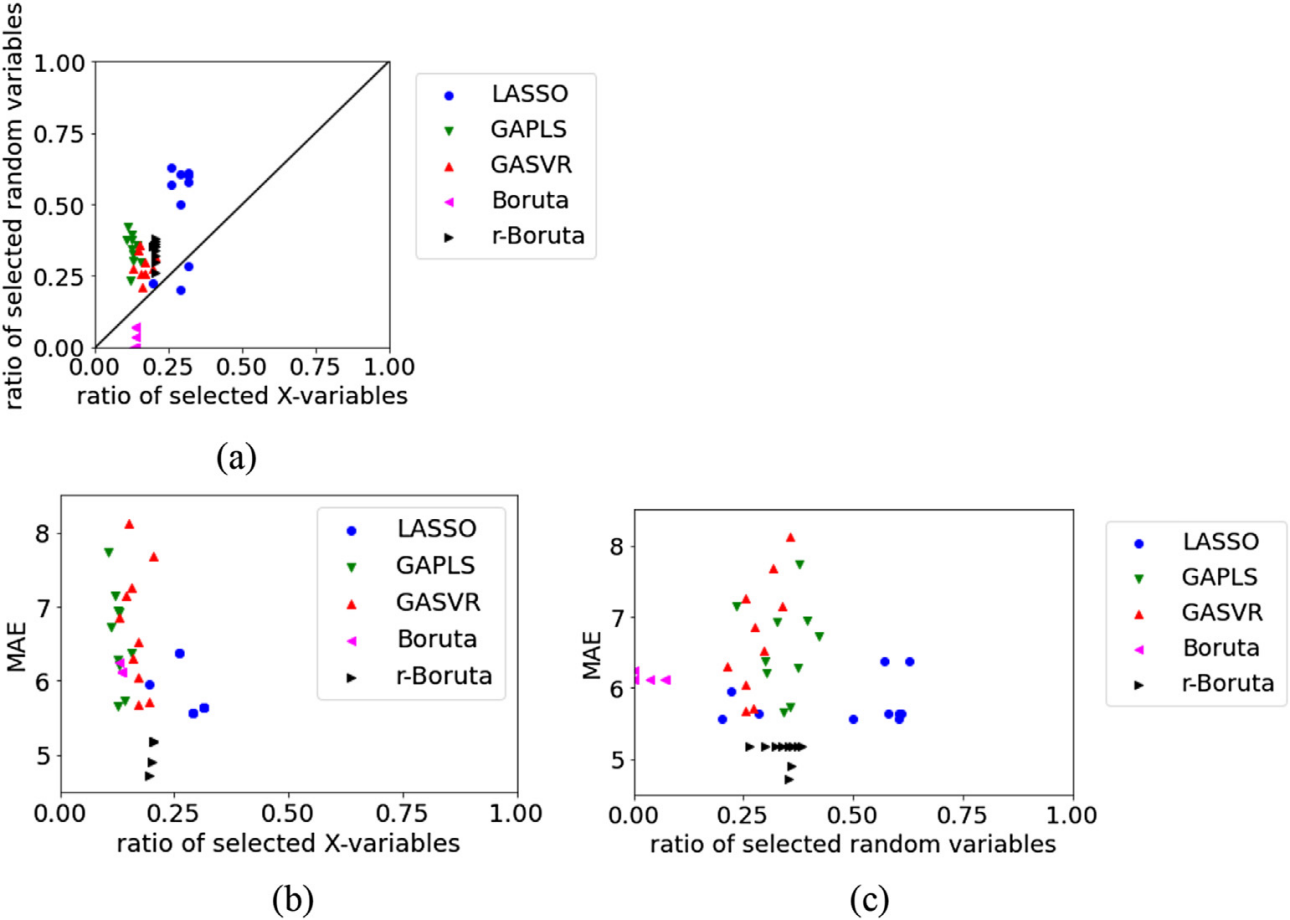
Relationships between the MAE, the ratio of selected X, and ratio of selected random variables for the test data in BP data set using RDKit: (a) ratio of selected X versus ratio of selected random variables, (b) ratio of selected X versus MAE, (c) ratio of selected random variables versus MAE.

**Figure 2:**
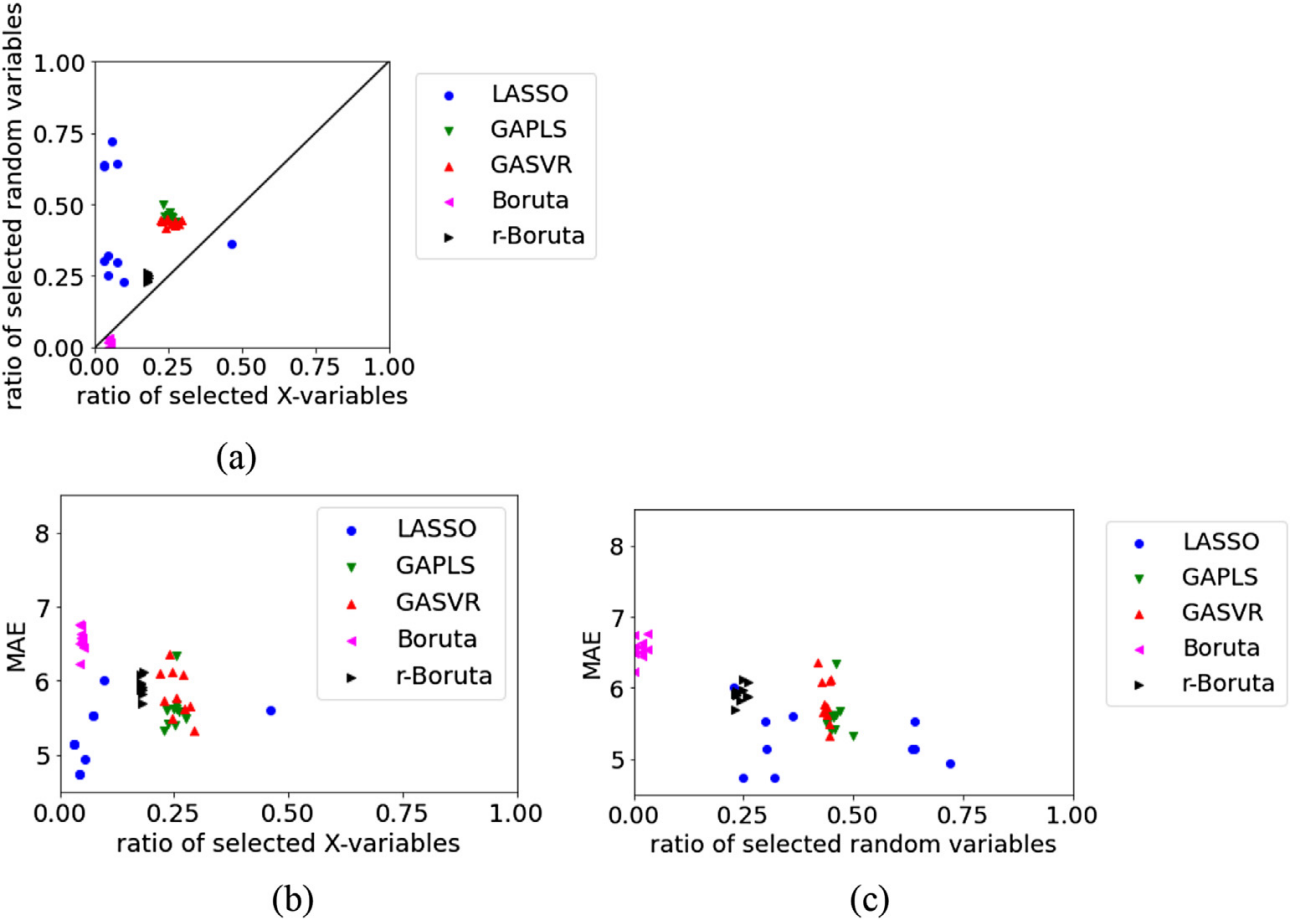
Relationships between the MAE, the ratio of selected X, and ratio of selected random variables for the test data in BP dataset using Mordred: (a) ratio of selected X versus ratio of selected random variables, (b) ratio of selected X versus MAE, (c) ratio of selected random variables versus MAE.

**Figure 3:**
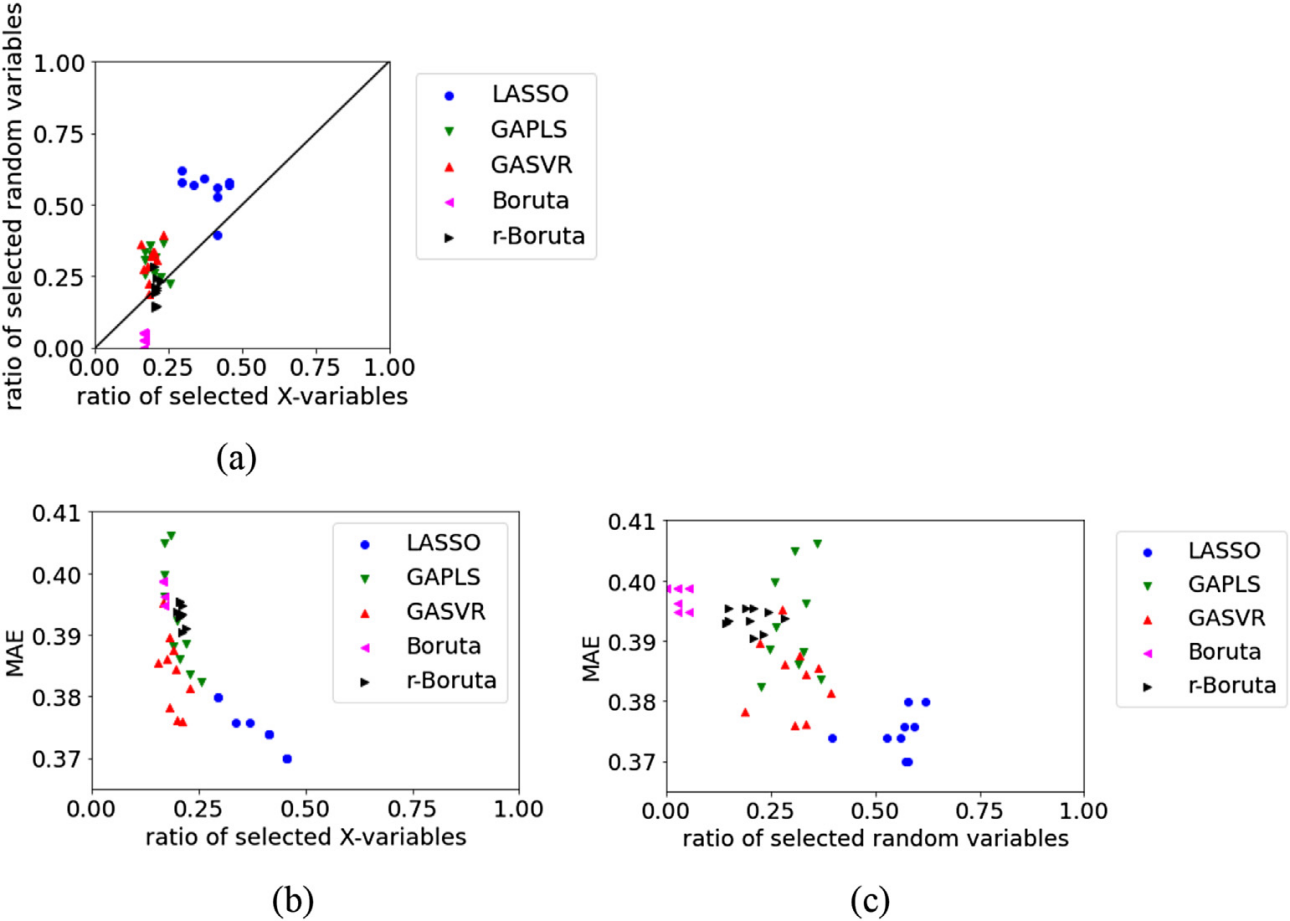
Relationships between the MAE, the ratio of selected X, and ratio of selected random variables for the test data in logS dataset using RDKit: (a) ratio of selected X versus ratio of selected random variables, (b) ratio of selected X versus MAE, (c) ratio of selected random variables versus MAE.

**Figure 4:**
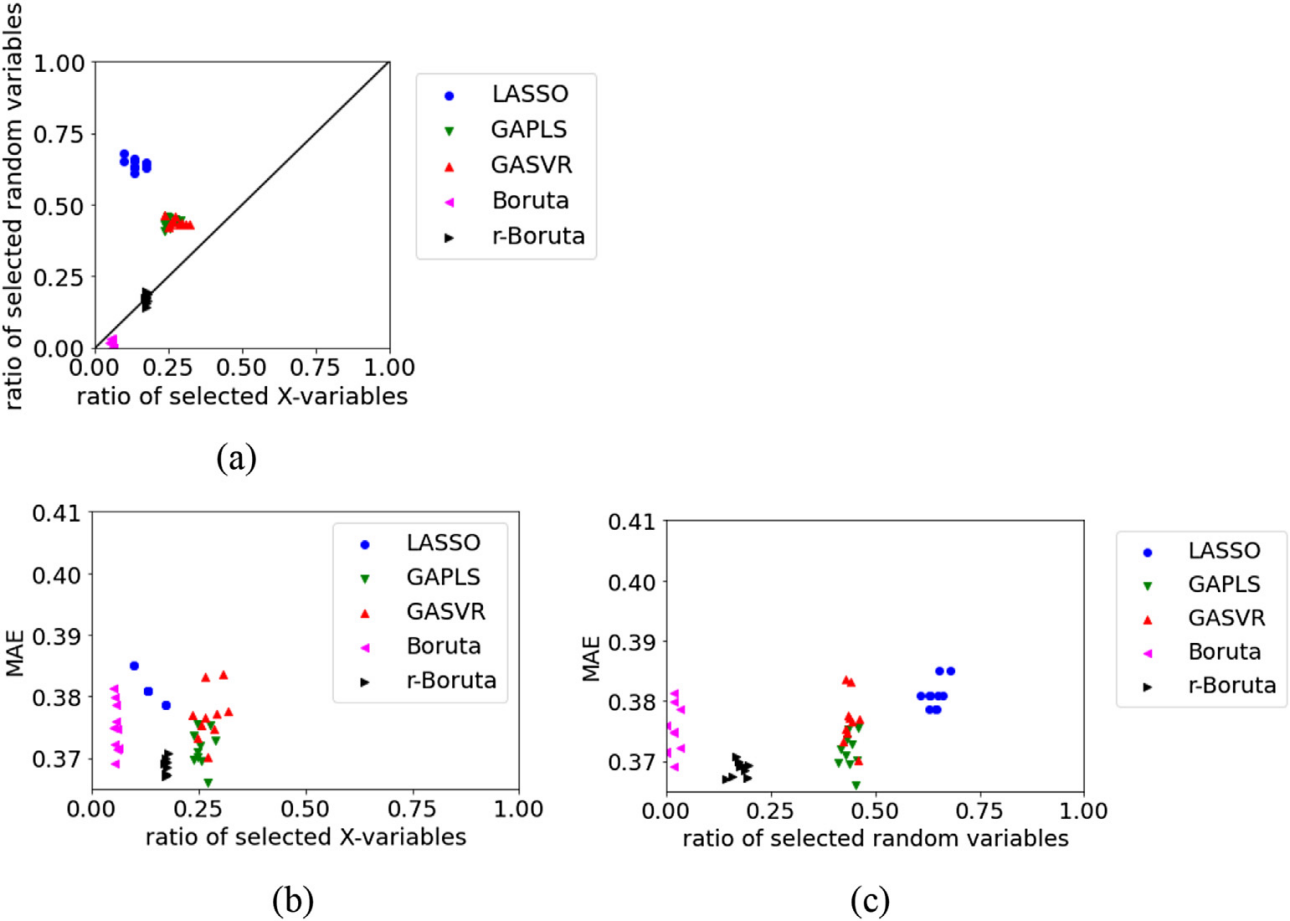
Relationships between the MAE, the ratio of selected X, and ratio of selected random variables for the test data in logS dataset using Mordred: (a) ratio of selected X versus ratio of selected random variables, (b) ratio of selected X versus MAE, (c) ratio of selected random variables versus MAE.

**Figure 5:**
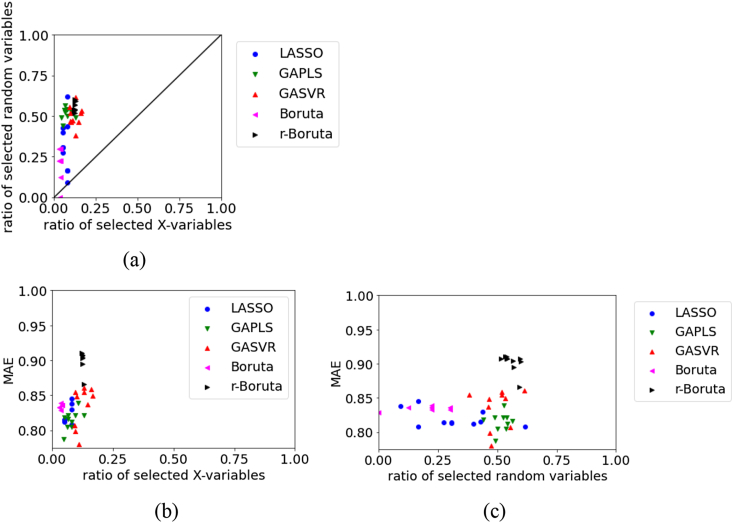
Relationships between the MAE, the ratio of selected X, and ratio of selected random variables for the test data in pIC_50_ dataset using RDKit: (a) ratio of selected X versus ratio of selected random variables, (b) ratio of selected X versus MAE, (c) ratio of selected random variables versus MAE.

**Figure 6:**
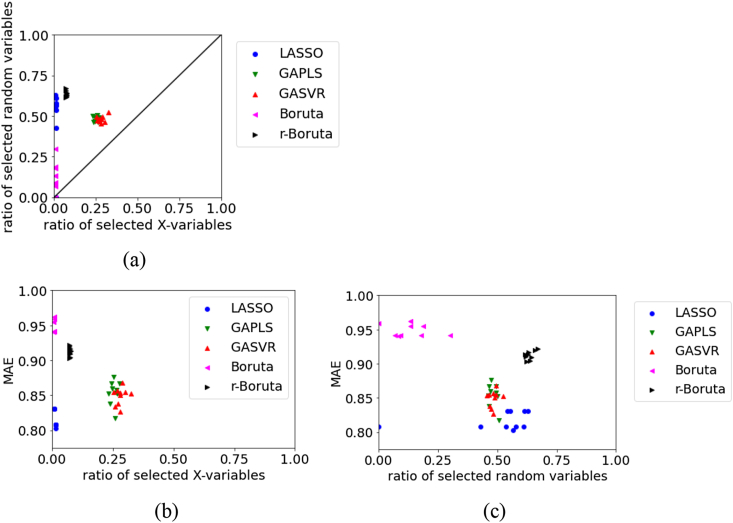
Relationships between the MAE, the ratio of selected X, and ratio of selected random variables for the test data in pIC_50_ dataset using Mordred: (a) ratio of selected X versus ratio of selected random variables, (b) ratio of selected X versus MAE, (c) ratio of selected random variables versus MAE.

**Figure 7:**
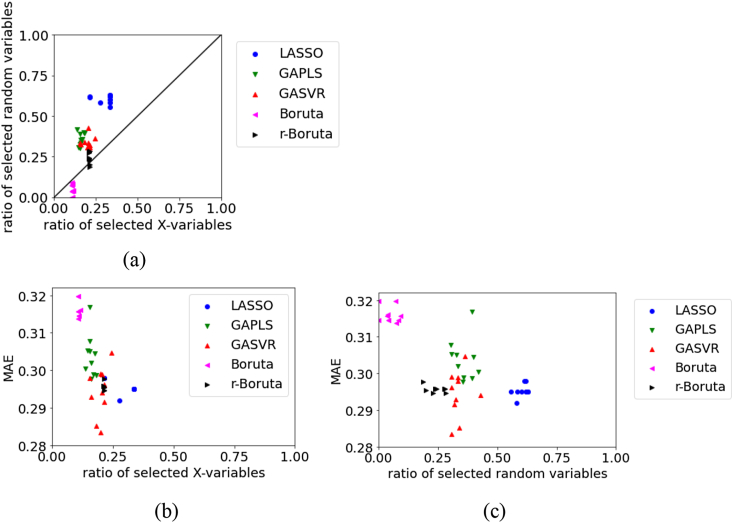
Relationships between the MAE, the ratio of selected X, and ratio of selected random variables for the test data in pIGC_50_ dataset using RDKit: (a) ratio of selected X versus ratio of selected random variables, (b) ratio of selected X versus MAE, (c) ratio of selected random variables versus MAE.

**Figure 8:**
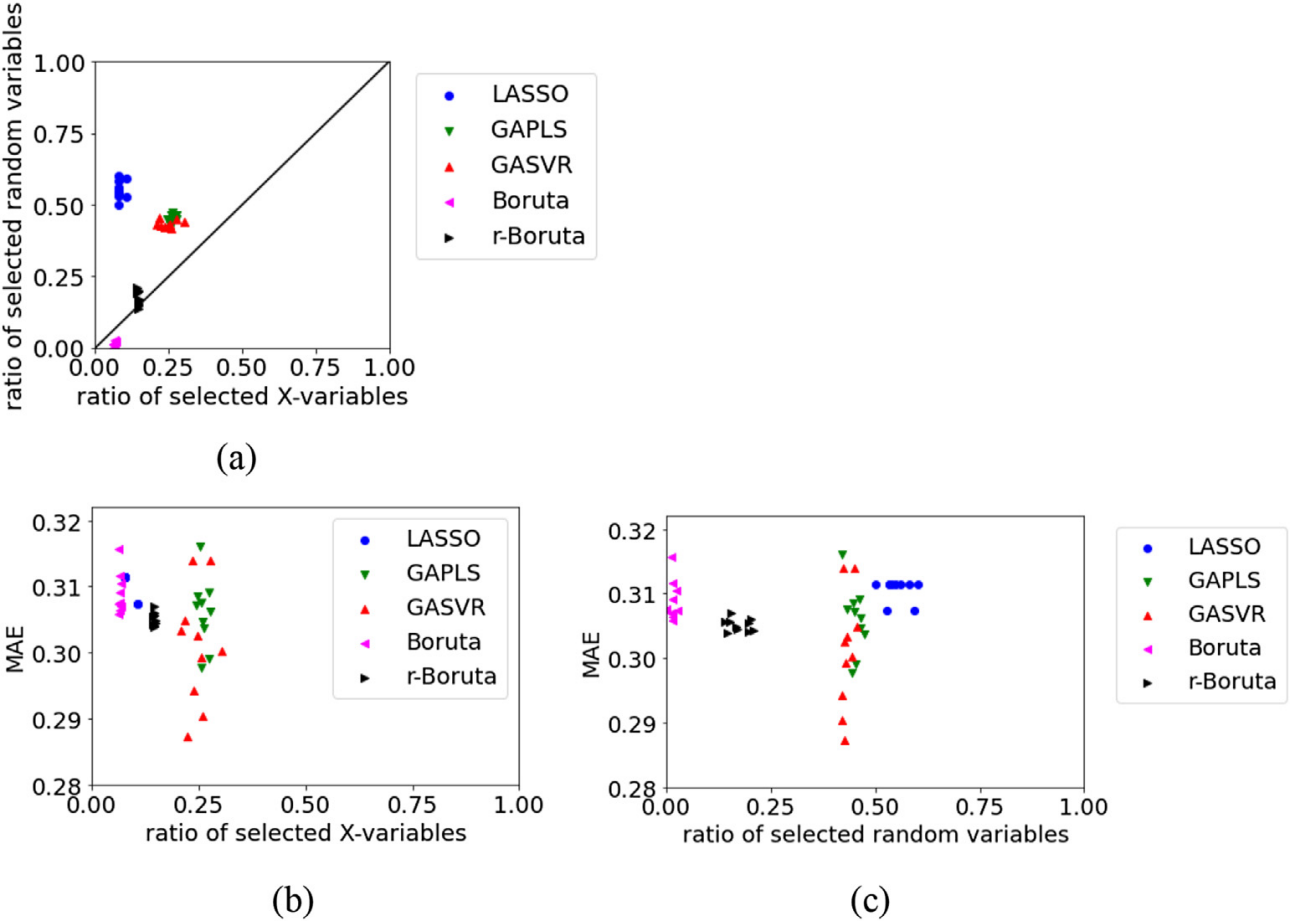
Relationships between the MAE, the ratio of selected X, and ratio of selected random variables for the test data in pIGC_50_ dataset using Mordred: (a) ratio of selected X versus ratio of selected random variables, (b) ratio of selected X versus MAE, (c) ratio of selected random variables versus MAE.

**Figure 9:**
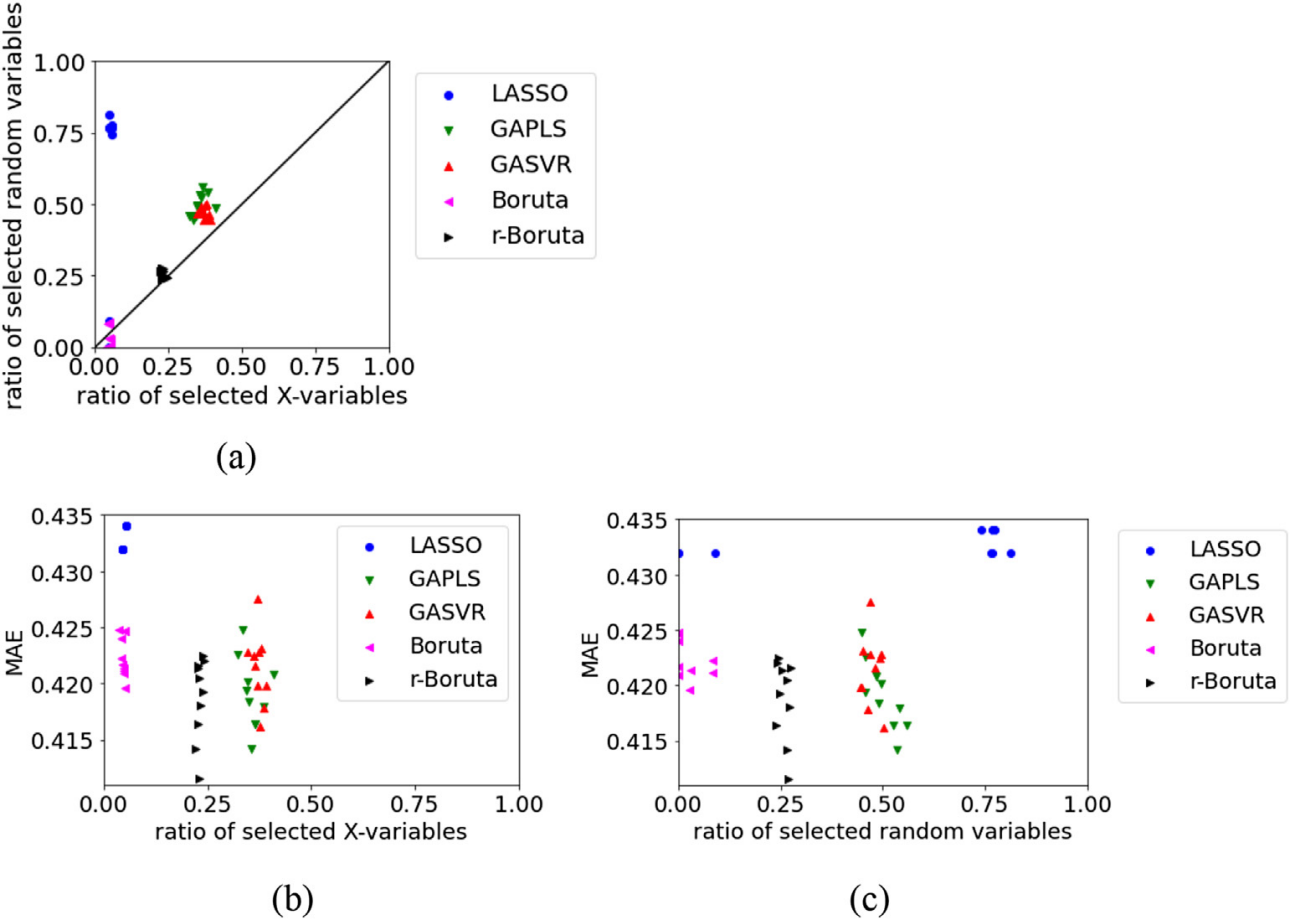
Relationships between the MAE, the ratio of selected X, and ratio of selected random variables for the test data in API1 dataset: (a) ratio of selected X versus ratio of selected random variables, (b) ratio of selected X versus MAE, (c) ratio of selected random variables versus MAE.

**Figure 10:**
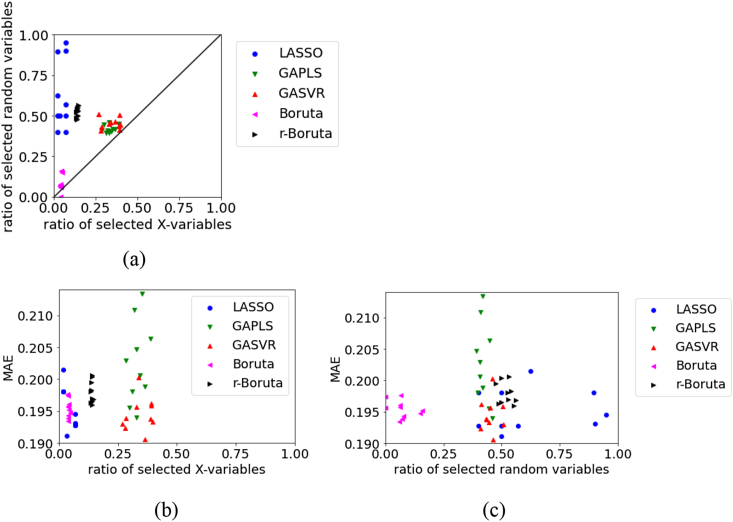
Relationships between the MAE, the ratio of selected X, and ratio of selected random variables for the test data in API2 dataset: (a) ratio of selected X versus ratio of selected random variables, (b) ratio of selected X versus MAE, (c) ratio of selected random variables versus MAE.

**Figure 11:**
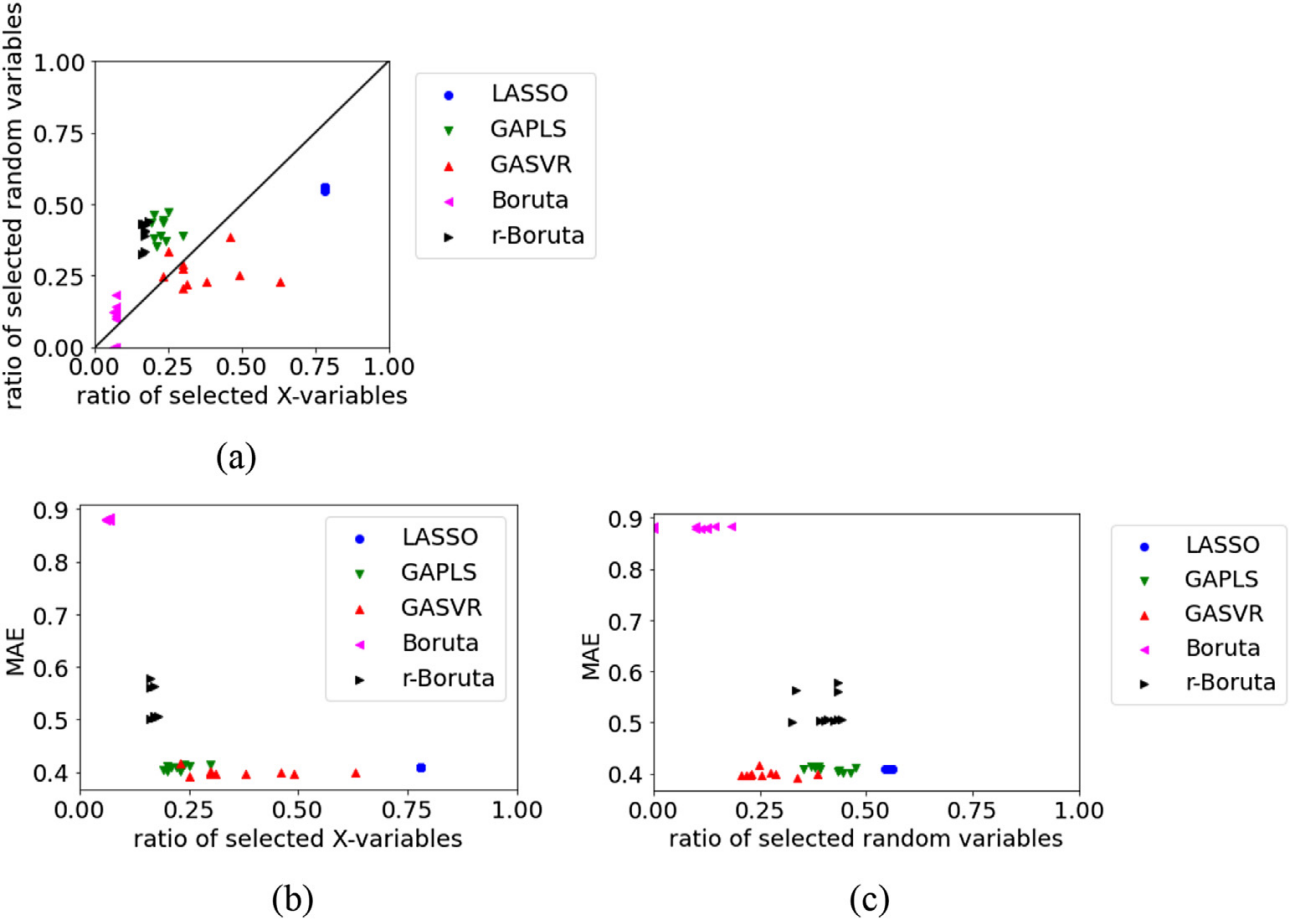
Relationships between the MAE, the ratio of selected X, and ratio of selected random variables for the test data in Wheat dataset: (a) ratio of selected X versus ratio of selected random variables, (b) ratio of selected X versus MAE, (c) ratio of selected random variables versus MAE.

**Figure 12:**
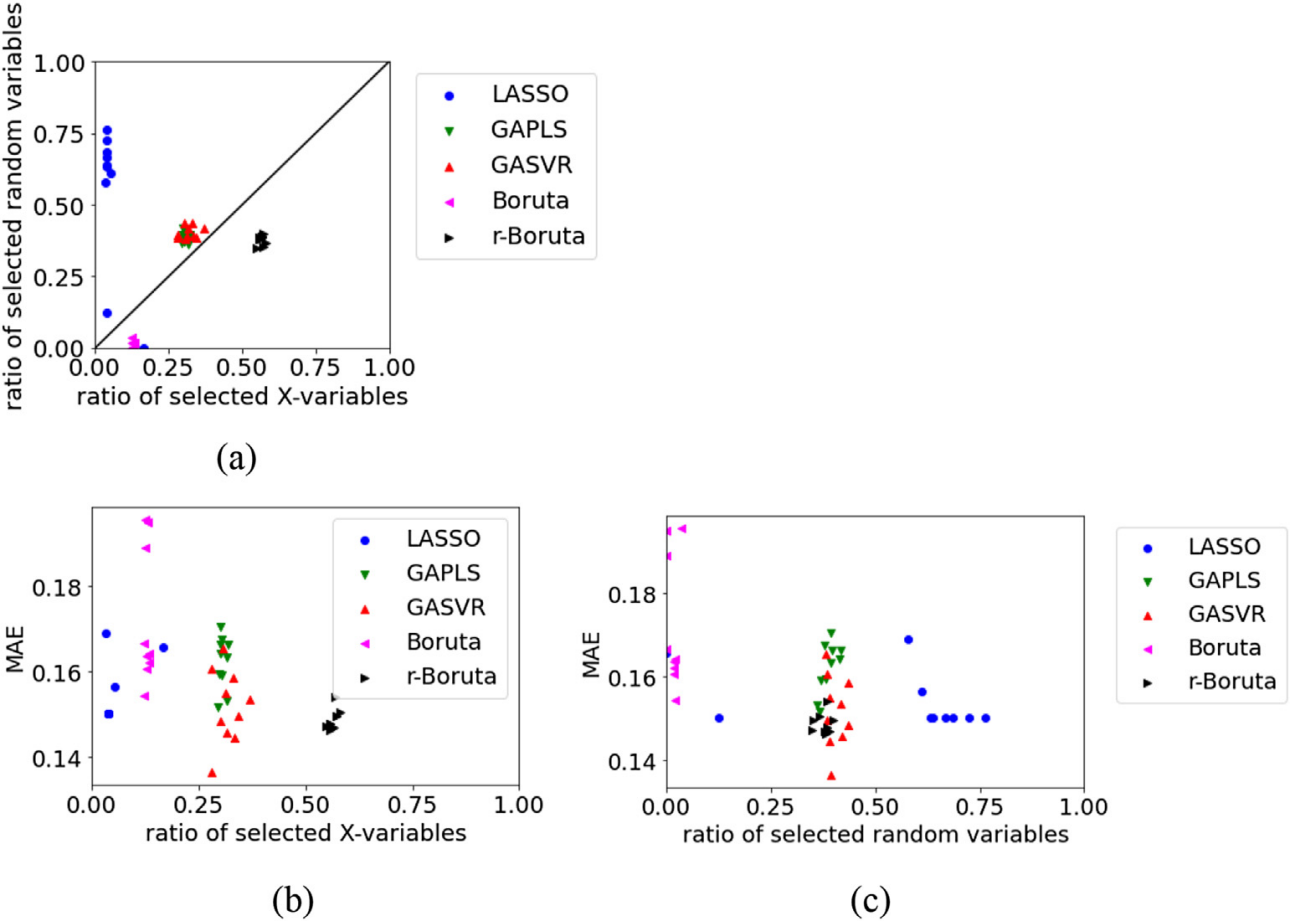
Relationships between the MAE, the ratio of selected X, and ratio of selected random variables for the test data in Gasoline dataset: (a) ratio of selected X versus ratio of selected random variables, (b) ratio of selected X versus MAE, (c) ratio of selected random variables versus MAE.

Figures 1, 2, 3, 4, 5, 6, 7, 8, 9, 10, 11, 12 show relationships of the MAE, the ratio of selected original X, and ratio of selected random variables for each dataset. For the test data in BPs using RDKit, Figure 1 illustrates that in LASSO, the numbers of selected original X and selected random variables are large, whereas in Boruta, the number of selected random variables tends to be small. Compared with LASSO, Boruta, and r-Boruta, the variability of the MAE is larger in GAPLS and GASVR. GAPLS and GASVR are considered to have a significant effect on the estimation accuracy of the initial value in the GA. In Boruta and r-Boruta, the variability in the results is smaller compared with that in LASSO, GAPLS, and GASVR. We believe that stable variable selection can be achieved through Boruta and r-Boruta. The numbers of selected original X and random variables in r-Boruta tend to be larger than those in Boruta; however, the MAE is smaller and the estimation accuracy is greater in r-Boruta. Although there is a greater possibility of variables unrelated to physical properties being selected in r-Boruta than in Boruta, it is less likely for important variables related to physical properties to be deleted in r-Boruta. Even if r-Boruta selects variables unrelated to physical properties, the estimation accuracy does not decrease according to the method used in the subsequent regression analysis.

For the test data in BPs using Mordred, as shown in Figure 2, the number of selected random variables is larger in LASSO and smaller in Boruta, but LASSO has a smaller MAE. Across all variable selection methods, the MAE tends to increase when the number of selected random variables is small. In variable selection, it is important not to omit important variables that are related to physical properties, and even when variables unrelated to physical properties are selected, a regression model with high estimation accuracy can be constructed using various regression analysis methods. The variability in the results of variable selection by the Boruta and r-Boruta approaches is smaller than that by the LASSO, GAPLS, and GASVR approaches. Although r-Boruta has a larger number of selected original X and selected random variables than Boruta, its MAE is smaller and the prediction accuracy is higher.

For the test data in logS using RDKit, Figure 3 shows that the number of selected random variables is large in LASSO and small in Boruta. There are large variations in the results of variable selection and MAE in GAPLS and GASVR. However, in terms of variable selection, when the number of the selected original X is large, the MAE tends to be small even when the number of selected random variables is large. In terms of estimation accuracy, it is important not to delete variables related to physical properties in variable selection. Although the MAE tends to be small when the number of selected random variables is large, in reality, the MAE is believed to be related to the number of selected original X. This makes the relationship between the number of selected random variables and the MAE a pseudo-correlation.

Figure 4 illustrates similar details for the test data in log S using Mordred. As shown in the figure, the number of selected random variables was large with LASSO and small with Boruta and r-Boruta. Although both the number of selected original X and selected random variables were larger with r-Boruta than with Boruta, its MAE was smaller and prediction accuracy was higher. The use of r-Boruta may have prevented the exclusion of important variables related to physical properties. The variation in MAE is large for GAPLS and GASVR. However, in approaches other than Boruta, the MAE tended to be larger when the number of selected random variables was large. As the number of X with Mordred is large, the selection of a large number of variables unrelated to physical properties may have reduced the prediction accuracy of the regression model.

Figure 5 shows the results from the test data in pIC_50_ using RDKit. The figure illustrates that the variability, in the results of variable selection, was large across all variable selection methods, and the number of selected random variables tended to be large for Boruta and r-Boruta. We believe that there were variations in the results of the variable selection due to the small number of samples. Furthermore, MAE tended to be larger with r-Boruta than with Boruta. Here, it is apparent that the number of selected random variables became larger after an improper setting of r, while the number of samples was small, which reduced the prediction accuracy of the regression model.

For the test data in pIC_50_ using Mordred, Figure 6 illustrates that the number of selected random variables tends to be large for all variable selection methods. This is probably because the number of samples is small and the number of variables is large, making it more likely that random variables that are accidently correlated with activity would be selected. The number of selected random variables is larger in r-Boruta than in Boruta. However, the MAE is smaller in r-Boruta, and the prediction accuracy is higher. The MAE is smaller in LASSO. As the number of samples is small and the number of variables is large, we believe that the nonlinear method is prone to overfitting, whereas the linear method of LASSO prevents overfitting.

The number of selected random variables is large in LASSO and small in Boruta and r-Boruta for the test data in pIGC_50_ using RDKit, as shown in Figure 7. Even though the number of selected random variables is larger in r-Boruta than in Boruta, the MAE is smaller and prediction accuracy is higher in r-Boruta. We believe this is because the exclusion of important variables related to activity is prevented. Additionally, the variability in the results of variable selection and results of the MAE is larger in GAPLS and GASVR. However, in all methods of variable selection, the MAE generally tends to decrease when the numbers of selected original X and selected random variables increases. It is important to note that variables related to y are not excluded, and even if there are variables unrelated to y, various regression analysis methods can be used to increase the estimation accuracy of the regression model.

For the test data in pIGC_50_ using Mordred, Figure 8 illustrates that the number of selected random variables is large in LASSO and small in Boruta and r-Boruta. Although r-Boruta has a larger number of selected original X and random variables compared with Boruta, its MAE is smaller and the prediction accuracy is higher. We believe that with r-Boruta, the fact that variables related to *y* are not excluded helps improve the prediction accuracy of the regression model by regression analysis methods even when variables unrelated to y are present. The variability in the MAE is larger in GAPLS and GASVR. As the number of X is large, it is more susceptible to influence from the initial value in the GA.

The variable relationships of the test data for API1 are shown in Figure 9. As shown in the figure, the number of selected original X is the same between the LASSO and Boruta approaches, but the number of selected random variables is larger in LASSO. This is believed to have resulted in the selection of variables unrelated to y, which makes the MAE larger and the prediction accuracy lower in LASSO than in Boruta. The numbers of selected original X and selected random variables are larger in r-Boruta than in Boruta. As Boruta has a smaller MAE and higher prediction accuracy of the regression model than r-Boruta, we believe that the non-exclusion of variables related to y is more important to the prediction accuracy of the regression model than the inclusion of variables unrelated to y.

For the test data in API2, Figure 10 illustrates that the number of selected random variables is large in LASSO and small in Boruta. The fact that the variability in the MAE is large in GAPLS is apparently caused by the differences in the initial values of the GA as a result of the small number of samples. Despite the MAE in Boruta being similar to that of other variable selection methods, both the number of selected original X and number of selected random variables help construct a compact model that is smaller than that achieved by other variable selection methods.

For the test data in Wheat, Figure 11 illustrates that the number of selected original X and number of random variables are large in LASSO and small in Boruta. However, the MAE tends to be smaller, and the prediction accuracy of the regression model tends to be higher with a larger number of selected variables. The MAE tends to be small, even when the number of selected random variables is large. This is thought to be because the number of selected original X is large when the number of selected random variables is large. The number of selected random variables was larger with Boruta than with r-Boruta, but the MAE tended to be smaller, and the prediction accuracy of the regression model tended to be better with Boruta. In variable selection, we can say that not deleting variables related to y is more important to the accuracy of the regression model than selecting variables unrelated to y.

The variability in the MAE is large in the LASSO, GAPLS, GASVR, and Boruta approaches for the test data in Gasoline, as shown in Figure 12. This may be attributable to the small sample size. Even in this situation, the results of r-Boruta remain stable. We believe that it is important to set an appropriate threshold for the percentiles in the Boruta approach. When the number of selected variables is large, the MAE tends to be small. Not deleting variables related to *y* can be considered an important factor in variable selection.

## 4 Conclusions

In QSAR/QSPR, it is difficult to discuss the performance of variable selection methods because the predictive performance of regression models using the selected X depends on regression analysis methods. Furthermore, because one of the purposes of variable selection methods is to increase the interpretability of the regression model between chemical structures and activities/properties, it is desirable to select fewer variables and remove unnecessary variables. Therefore, in this study, the performance of variable selection methods was discussed in terms of multiple metrics: predictive performance of regression models, proportion of selected variables, and proportion of selected random variables in QSAR/QSPR. In this study, the variable selection performance of the LASSO, GAPLS, GASVR, Boruta, and r-Boruta methods, which were established based on the pseudo-correlation of the percentile threshold in Boruta, was investigated. The investigation was performed with respect to the prediction error of the regression model, number of selected original X, and number of selected random variables. The prediction errors in the regression model were evaluated using 24 regression analysis methods. Thus, although variable selection methods are well-established and being developed, it is possible to discuss variable selection methods in depth by validating the methods in terms of multiple metrics, as proposed in this paper. It was confirmed that there is no best variable selection method for all the three metrics and that each method has its own characteristics.

The results showed that the number of selected original X and the number of random variables were large in LASSO and small in Boruta. GAPLS, GASVR, and r-Boruta fell between these two methods in terms of these parameters. However, the prediction error of a regression model does not necessarily increase with a larger number of selected random variables. In r-Boruta, the number of selected random variables was larger than that with Boruta, but the prediction error of the regression model tended to be smaller. Even if X unrelated to y is selected by variable selection, a regression model with good accuracy that ignores the influence of such noise variables can be constructed by applying various regression analysis methods. Thus, even if X unrelated to y is selected by variable selection, it is important that X related to y is not deleted.

Although the results of variable selection and the MAE were stable in both Boruta and r-Boruta, the variability in the MAE was particularly large in methods such as GAPLS and GASVR. We believe that the results were stable in Boruta because its calculation process incorporates a testing and verification step, whereas the results of GAPLS and GASVR were unstable because of their dependency on the initial value in the GA. When the number of samples was small, random variables tended to be more commonly selected by variable selection methods, thereby making the MAE results unstable. We believe that the LASSO results in this situation were stable because it is a linear regression approach for providing an analytical solution.

When the number of samples was large, such as with logS and pIGC50, RDKit tended to have a small MAE when the number of selected X was large; this is likely due to the small number of variables. The MAE tended to be small when the number of selected random variables was large; however, the number of selected variables is an important factor in reality. Moreover, a pseudo-correlation exists between the number of selected random variables and the MAE. In Mordred, the MAE tended to be large when the number of selected random variables was large, presumably because of the large number of features, and it is thought to be due to the influence of random variables.

As the numbers of selected X and random variables were small in Boruta, it can be effective for interpreting the results of variable selection. However, due to the possibility that variables related to y are deleted and the prediction accuracy of the regression model decreases, care is required when using this approach. Such risks can be reduced by setting a percentile threshold for Boruta based on pseudo-correlation. The number of selected X was large in LASSO; this can reduce the likelihood of X related to y being deleted. However, as many X unrelated to y are also selected, it is difficult to interpret the results of variable selection.

Therefore, our examination of variable selection was diverse and detailed, through the evaluation of variable selection methods based on the prediction error of the regression model, number of selected original X, and number of selected random variables. The discussion in this paper is expected to promote the development of variable selection methods in the future.

## Declarations

### Author contribution statement

Hiromasa Kaneko: Conceived and designed the experiments; Performed the experiments; Analyzed and interpreted the data; Contributed reagents, materials, analysis tools or data; Wrote the paper.

### Funding statement

This work was supported by the 10.13039/501100001691Japan Society for the Promotion of Science (JP19K15352).

### Data availability statement

Data included in article/supp. material/referenced in article.

### Declaration of interests statement

The authors declare no conflict of interest.

### Additional information

No additional information is available for this paper.
